# Experimental Study on Erosion–Corrosion of TP140 Casing Steel and 13Cr Tubing Steel in Gas–Solid and Liquid–Solid Jet Flows Containing 2 wt % NaCl

**DOI:** 10.3390/ma12030358

**Published:** 2019-01-24

**Authors:** Jiarui Cheng, Zhen Li, Ningsheng Zhang, Yihua Dou, Lu Cui

**Affiliations:** 1 Department of Mechanical Engineering, Xi’an Shiyou University, Xi’an 710065, China; lizhen@xsyu.edu.cn (Z.L.); cjr88112@stu.xjtu.edu.cn (Y.D.); cjr88112@126.com (L.C.); 2 Department of Petroleum Engineering, Xi’an Shiyou University, Xi’an 710065, China; nszhang@xsyu.edu.cn

**Keywords:** Gas–solid flow, liquid–solid flow, erosion–corrosion, three-electrode system, synergistic effect

## Abstract

To study the erosion–corrosion characteristics of TP140 casing steel and 13Cr tubing steel in oil fields, we performed gas–solid and liquid–solid jet flow experiments to control particle addition, jet angle, and flow velocity and measure erosion and corrosion components. Meanwhile, we used a standard three-electrode system to study the changes in electrochemical parameters on a metal surface in a two-phase flow containing 2 wt % NaCl. Results showed that erosion is mainly dominated by the flow velocities and impact angles of particles, and corrosion rate is mainly affected by liquid flow rate. The erosion rates of the two materials increase with flow velocity, and the critical angle of maximum erosion rate exists. Meanwhile, flow velocity growth increases the current density on the TP140 surface while reducing the corrosion potential of 13Cr, but the effect of the angle on the two parameters is relatively small. The uniform corrosion of TP140 increases the erosion rate in the range of 10–20%, and the pitting of 13Cr increases the erosion rate in the range of 30–90%, indicating that the interaction between the erosion and corrosion of stainless steel is obvious.

## 1. Introduction

Tubing and casing in oil fields are subjected to the erosion–corrosion of high-velocity two-phase flow during fracturing (for a detailed description of fracturing technology, readers are referred to Ref. [1]), which decreases the strength of pipe walls and causes leakage accidents. Erosion damage usually refers to the phenomenon where solid particles are carried by a liquid or gas and impinged on a wall to cause material loss [2,3], and material loss due to electrochemical reaction is called electrochemical corrosion [4]. When particles are added to flowing electrolyte liquids, physical and electrochemical coupling damage to the wall occurs and changes with flow velocity, particle concentration, temperature, and pH. Therefore, studying the mechanism and influencing factors of erosion, corrosion, and the erosion–corrosion of metal walls has a significance in avoiding damage to pipelines and equipment.

The interactions between erosion and corrosion are complex, and both processes either can complement each other and accelerate material removal rate or may suppress total wear rate [5]. In a liquid–solid two-phase flow, the collision of particles with a wall causes the corrosion of product films, which peel off, thereby exposing the fresh metal surface and subsequently accelerating corrosion reactions [6]. Meanwhile, the corrosive surface easily falls off with physical cutting, which disrupts the generation of a hardened layer and impairs erosion protection. Stack [7,8,9,10] subdivided the erosion and corrosion components in this interaction process through experimental research. The total erosion–corrosion rate can be expressed as:

(1) $$K_{T}=K_{E}+K_{C}+\Delta K$$

or

(2) $$K_{T}={K^{\prime }}_{E}+{K^{\prime }}_{C}$$

where *K_E_* is the pure erosion rate, *K_C_* is the pure corrosion rate, Δ*K* is the synergistic value between erosion and corrosion, *K′_E_* is the erosion rate in liquid–solid flow, and *K′_C_* is the corrosion rate in liquid–solid flow. The erosion and corrosion rates can also be subdivided into:

(3) $${K^{\prime }}_{E}=K_{E}+\Delta K_{E}$$

and

(4) $${K^{\prime }}_{C}=K_{C}+\Delta K_{C}$$

where Δ*K_E_* is the corrosion-enhanced erosion rate, and Δ*K_C_* is the erosion-enhanced corrosion rate. Using this division method, many studies used liquid–solid or gas–solid experiments to obtain components in a NaCl or CO_2_ solution. Islam [11] designed a test setup for mitigating the limitations of the in situ method and obtained total material loss rate and the components of erosion, corrosion, and their synergistic interactions with API X-70. Their results showed that a significant correlation exists between erosion and corrosion, and erosion and corrosion enhances each other, each contributing to significant synergism. Yang [12] adopted a similar experiment to obtain parametric effects, including sand concentration, slurry flow velocity, and impact angle, on the erosion–corrosion of X65 steel in an oil sand slurry. Their test results showed that erosion is the dominant factor when the potential of steel is relatively negative at high flow velocities, and steel corrosion is important in the erosion–corrosion process at positive potentials. To investigate the behavior of high velocity oxy-fuel (HVOF) nickel aluminum bronze coatings under erosion, corrosion, and erosion–corrosion conditions, Tan [13] used the correlation between conventional mass loss measurements and electrochemical noise techniques to study the identification and quantification of synergistic effects. Their work demonstrated, for the first time, a possible correlation between the standard deviation ratios of electrochemical current noise and gravimetric data associated with erosion–corrosion. Sasaki [14] investigated the electrochemical reaction characteristics of 304L stainless-steel in liquid–solid two-phase flow and measured the open-circuit potential and polarization curve. The fluid flow alternatively impeded pitting corrosion by washing away the aggressive anolyte beneath the pit cover over the metastable pit mouth or by rupturing the cover mechanically. These typical studies for erosion–corrosion are basically based on comparative experiments and aimed at obtaining the variation among erosion and corrosion components. Some measures, including the addition of preservatives and control of liquid flow rates and surface coatings are typically used to determine erosion and corrosion rates.

In addition to the experimental research on erosion–corrosion, several studies have attempted to obtain the synergistic effect of erosion and corrosion in oxygen corrosion environment through theoretical calculation or numerical simulation. Stack [8] built a model of slurry erosion–corrosion processes for steel and considered the effects of increases in flow velocity, oxygen, and particle concentration. They constructed theoretical erosion–corrosion maps and determined the differences among erosion–corrosion mechanisms as a function of these variables. Zhang [15] took electrochemical measurements and performed computational fluid dynamics (CFD) simulation on micro-electrodes installed on an impingement jet system to study the flow-accelerated corrosion (FAC) of an X65 pipeline steel. By calculating the parameters of a local flow field, they were able to establish a link between flow and corrosion. These theoretical solutions and numerical analyses play an important role in the study of erosion–corrosion, but some key mechanisms cannot be obtained, such as fatigue, local corrosion, and roughness effects. Many experiments use the measurement results in pure water containing particles as pure erosion, which ignores the effects of oxygen corrosion and liquid forces on erosion.

TP140 casing and 13Cr tubing, which is a common string combination in oil fields, are widely used in high-temperature and high-pressure gas wells. The two kinds of tubing string are subjected to serious erosion and corrosion during high velocity liquid–solid flow containing chloride ion in fracturing. Therefore, we carried out erosion and corrosion experiments in gas–solid and liquid–solid jet environments for these two materials to obtain the variation rules of the factors and components of erosion–corrosion.

## 2. Experimental Method

A system containing gas–solid and liquid–solid two-phase flow sections was applied to the erosion and electrochemical corrosion characteristics of the TP140 material. The weight loss of the material under the impact of a gas–solid jet was used as the erosion rate, and the Tafel fitting value of the polarization curve measured by an electrochemical system was used as the corrosion rate in the liquid–solid flow. The programmable logic controller (PLC) screw feeder and the three-electrode electrochemical system were used together to ensure the accuracy of the test.

### 2.1. Pipe Flow System

**Figure 1:**
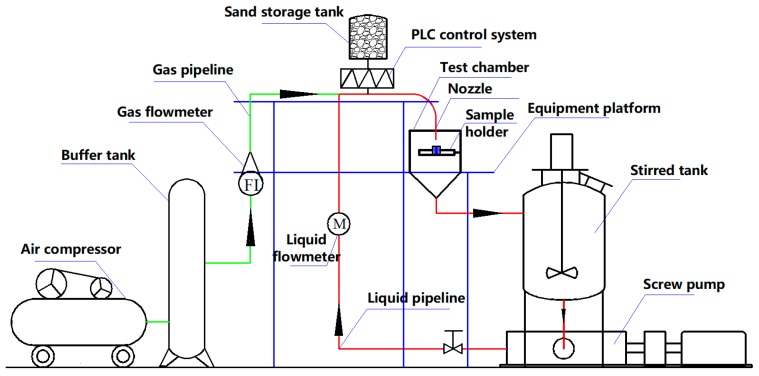
Gas–solid and liquid–solid experimental loop.

Figure 1 shows the gas–solid and liquid–solid experimental loop, including the screw pump, liquid flowmeter, test chamber, sample holder, stirred tank in the liquid flow system and air compressor, buffer tank, filter drier, gas flowmeter, sand storage tank, and PLC feeder in the gas flow system. In the gas–solid and liquid–solid experiments, particles were added during the flow process for the stabilization of particle concentration at 33 g/L. Once the particles collided with the sample surface, they were collected in the test section and the stirred tank because the sand would break after impact and affect the experimental results. The liquid pipeline was made of a 304-stainless-steel tube with a diameter of 40 mm, and a rubber hose with a diameter of 6 mm was used in the gas line. In this experiment, the total mass of sand was 30 kg, the total volume of liquid was 50 L, and the gas was dry air.

### 2.2. Test Section and Experimental Medium

**Figure 2:**
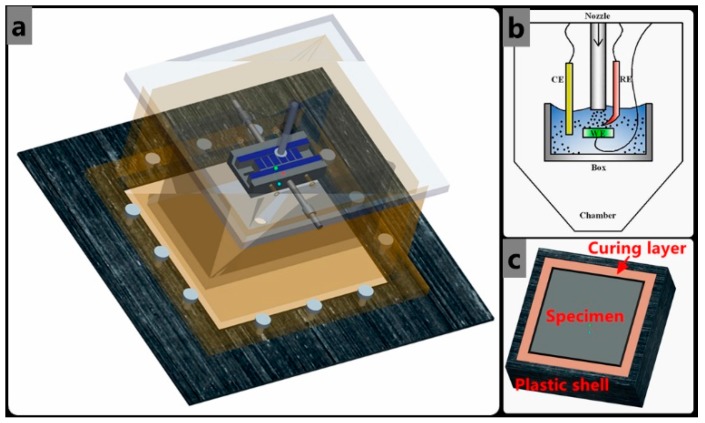
Schematic of the erosion–corrosion jet experimental system: (**a**) three-dimensional diagram of the test section, including the sample holder, the nozzle, and the peripheral glass shield; (**b**) installation diagram of three-electrode system; and (**c**) schematic of the sample package structure.

Electrochemical measurements were operated with a jet tester shown in Figure 2. A three-electrode electrochemical system was incorporated into the corrosion rig for open-circuit potential and potentiostatic testing. The saturated calomel reference electrode, which was placed in the test chamber, was connected to the standard three-electrode system. A long platinum wire was used as the counter electrode. For electrochemical monitoring, polarization curves were recorded by changing electrode potential at a sweep rate of 0.2 mV/s. The samples with cross-sections of 20 mm × 20 mm were made of TP140 casing and 13Cr tubing materials. The composition and mechanical properties are shown in Table 1 and Table 2, respectively. The exposed surface was sealed with epoxy resin and ground by using a 1200 grade SiC emery paper prior to installation. The pure erosion and corrosion rates were measured by the weight loss method, while the changed corrosion rate was measured by the electrochemical method in liquid–solid flow. A JSM-6390 stereo microscope (JEOL, Tokyo, Japan), which has a frame rate of 50 fr/s, was used for the documentation of the micro-erosion maps of the sample surface.

The experimental nozzle (length: 200 mm, inner diameter: 10 mm) was used at impact angles of 30°, 45°, 60°, and 90°. The velocities of the particles changed from 8 m/s to 20 m/s. The detailed conditions of the different experiments are shown in Table 3. Four jet angles and four flow velocities were designed for the comparison between the effects of particle impact angle and velocity on pure erosion and corrosion. Meanwhile, representative jet angles, including 45° and 90°, were selected for the comparison between the synergistic effects of erosion and corrosion under different particle impact patterns (i.e., extrusion and cutting).

## 3. Results

### 3.1. Gas-Solid Erosion (Pure Erosion)

**Figure 3:**
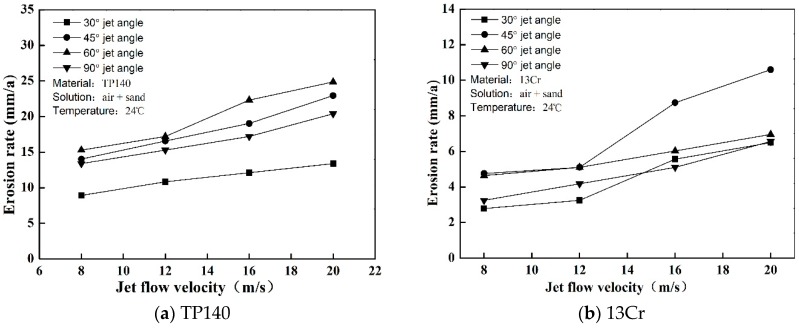
Erosion rate of TP140 and 13Cr under gas–solid jet flow.

The erosion of TP140 and 13Cr in the gas–solid flow were measured. The pure erosion rates of the material are shown in Figure 3 where they are expressed as annual thickness reduction (mm/a). The results show whether the erosion rate of TP140 or 13Cr is proportional to the jet flow velocity, which has been partially proven to be an exponential growth relationship [16]. According to the statistics of the factors affecting erosion [17], high hardness metals have strong erosion resistance. Thus, the erosion rate of TP140 is greater than that of 13Cr at each flow velocity. However, the relationship between impact angle and erosion rate is not a proportional relationship but a quadratic functional relationship. The maximum erosion rates of TP140 and 13Cr appear at 60° and 45°, respectively, and the minimum erosion rate of the two materials corresponds to an angle of nearly 30°.

**Figure 4:**
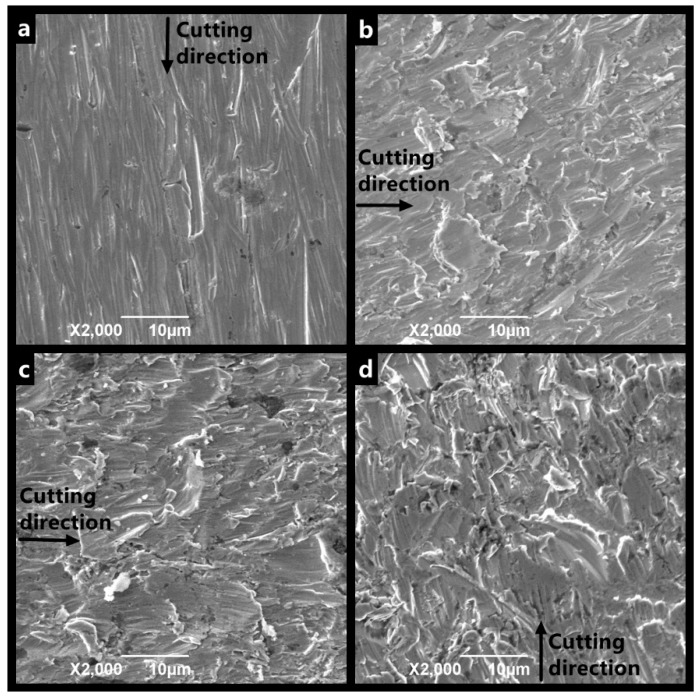
SEM images of 13Cr eroded at a jet velocity of 8 m/s: pure erosion at impact angle of (**a**) 30°, (**b**) 45°, (**c**) 60°, and (**d**) 90°.

The SEM images of 13Cr under different jet flow angles (Figure 4) show that the erosion surface can be divided into two types, namely, cutting and extrusive surfaces. The cutting surface is eroded at low impact angles, with the occasional impact of few particles, and shows uniform thinning (Figure 4a). The extrusive surface refers to the surface that is subjected to repeated particle impacts at a high angle and has craters, platelets, and extruding lips on its surface (Figure 4d). These lips are squeezed by constant deformation and are eventually stripped by subsequent particles. Many scratches, which are caused by the small-angle particle impacts, have the same direction all over the sample surface. Unlike extruding deformed lips, cutting lips mainly exist at the tip of the scratch and are more easily stripped. Deformation damage has a deep impact crater and many extruding materials, whereas cutting removal damage can peel off materials separately and leads to a long but shallow scratch.

### 3.2. Flow Induced Corrosion (Pure Corrosion)

**Figure 5:**
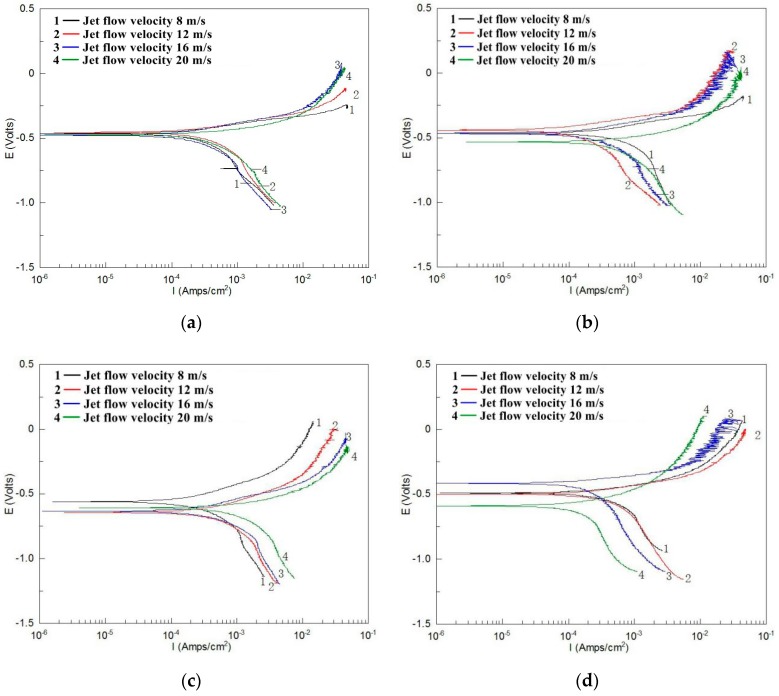
Polarization curves of TP140 within an angle of 30° up to 90° (2 wt % NaCl + distilled water). (**a**) 30° jet angle, (**b**) 45° jet angle, (**c**) 60° jet angle, and (**d**) 90° jet angle.

**Figure 6:**
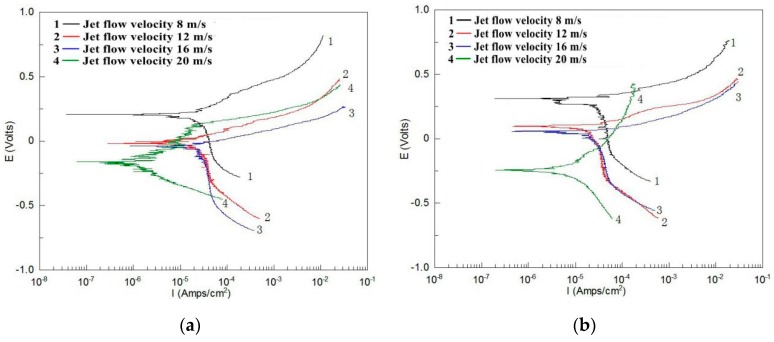

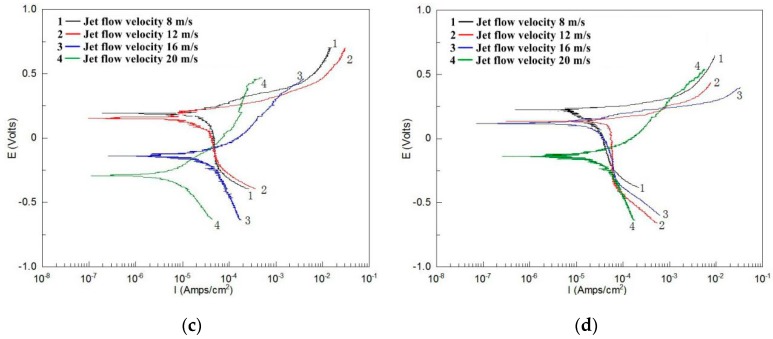
Polarization curves of 13Cr within an angle of 30° up to 90° (2 wt % NaCl + distilled water). (**a**) 30° jet angle, (**b**) 45° jet angle, (**c**) 60° jet angle, and (**d**) 90° jet angle.

Figure 5 and Figure 6 show the polarization curves of TP140 and 13Cr in the liquid solution. The results show an actively controlled anodic process and a mass transfer-controlled cathodic process for TP140 and 13Cr. Meanwhile, most corrosion potentials decrease to negative values as the flow velocity increases, which indicates that corrosion is more likely to occur at high flow velocities. As for the polarization curves of TP140 (Figure 5), no significant change in the slope of the anodic and cathodic curves is observed, but the measured value changes to a small potential and a large current density with increased flow velocity. The corrosion potential and current density for TP140 are sensitive to changes in flow velocity at small impact angles. Meanwhile, the increase of corrosion current density is more obvious from an angle of 45–60°. Unlike the regular changes in the polarization curves of TP140, the changes in the polarization curves of 13Cr at different angles are diverse (Figure 6). The corrosion potential changes obviously with increasing flow velocity, even reaching 0.52 V at an angle of 60°. Affected by chloride ions in the solution, no obvious passivation zone is observed in the anode region, and the slope of the cathodic polarization curve is significantly reduced. This indicates that the increase of flow velocity transforms the electrochemical reaction of the 13Cr surface from concentration polarization to activation polarization.

**Figure 7:**
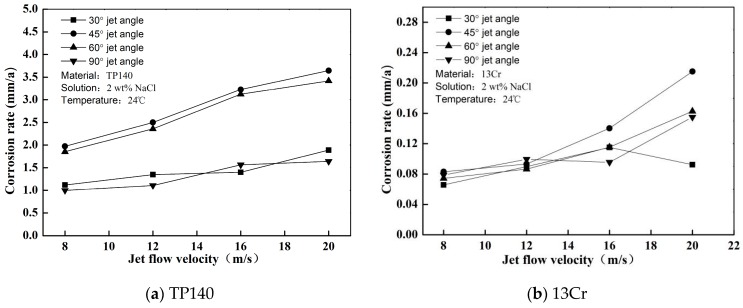
Pure corrosion rate for TP140 and 13Cr steel.

Similar to the trend of erosion rate in gas–solid flow, the pure corrosion rate also increases with the flow velocity as shown in Figure 7. The corrosion rate increase reaches 80% for TP140 and 168% for 13Cr when the flow velocity increases from 8 m/s to 20 m/s. These trends reflect that 13Cr stainless-steel is sensitive to the changes in the flow velocity of chloride-containing solutions. In addition, for both 13Cr and TP140, the jet angle corresponding to the maximum corrosion rate appears at a 45° jet angle, and a phenomenon exists where the corrosion rate within an angle of 30° to 45° is significantly greater than those at 30° and 90° for TP140.

### 3.3. Synergism of Erosion and Corrosion

**Figure 8:**
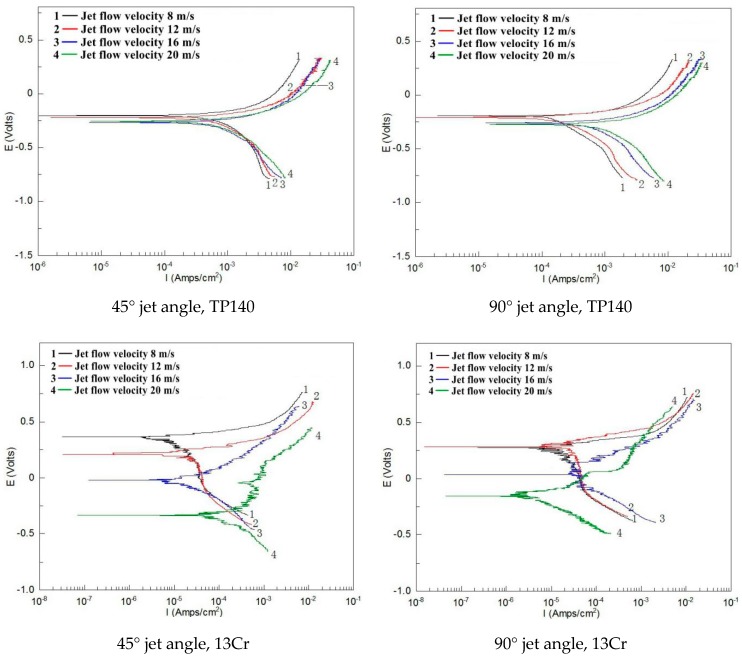
Polarization curves of TP140 and 13Cr with angles 45° and 90° (2 wt % NaCl + sand).

Based on the results of gas–solid erosion, namely, cutting and extrusive surfaces, an experiment is conducted on TP140 and 13Cr within an angle of 30–90° to compare with the results of the gas–solid and liquid experiments. Several representative polarization curves of the two materials at angles 45° and 90° are shown in Figure 8. The corrosion current density of TP140 at 90° is increased by approximately an order of magnitude with the increasing velocity from 8 m/s to 20 m/s, compared to only 20% in the liquid. In addition, for the polarization curves of 13Cr, the reduction in corrosion potential reaches 0.67 V at 45°, which is 29% higher than that in the liquid flow.

The pure erosion rate (*K_E_*), the pure corrosion rate (*K_C_*), the erosion rate in liquid–solid flow (*K′_E_*), the corrosion rate in liquid–solid flow (*K′_C_*), the corrosion-enhanced erosion rate (Δ*K_E_*), the erosion-enhanced corrosion rate (Δ*K_C_*), and the synergistic value between erosion and corrosion (Δ*K*) at the angles of 45° and 90° are shown in Table 4. In the comparison between *K′_E_* and *K_E_* at different jet flow angles, the increase in the erosion rate is less than 15% for TP140 and 30% for 13Cr. These results indicate that the erosion–corrosion of 13Cr is more sensitive to jet flow angle than that of TP140 in the liquid–solid flow. The difference between *K′_C_* and *K_C_* for TP140 is less than that for 13Cr, which means that the effects of particle impact on 13Cr is greater than that on TP140 in liquid–solid flow. The interaction amount (Δ*K*) indicates that the synergy of TP140 is more sensitive to angle change than that of 13Cr. 

## 4. Discussion

**Figure 9:**
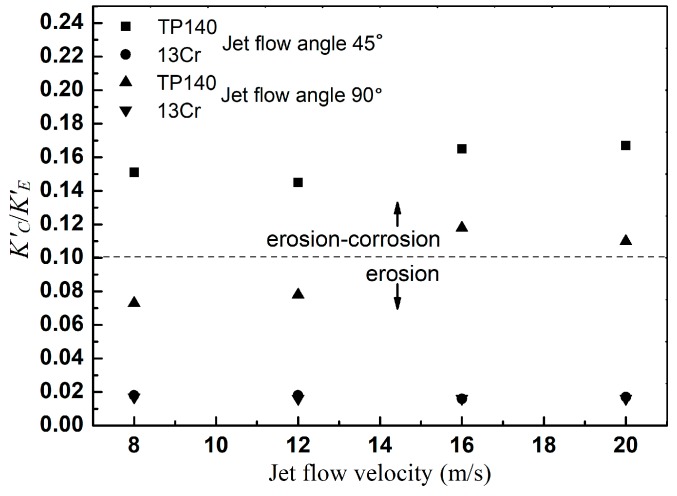
Map of dominant factors for TP140 and 13Cr.

According to the division of dominant factors in erosion–corrosion [7], as shown in Table 5, the map of dominant factors for TP140 and 13Cr is shown in Figure 9. The calculated results of *K′_C_*/*K′_E_* show that the material loss for the two metals can be classified as erosion–corrosion and erosion. The erosion–corrosion dominant factor means that corrosion has a significant effect on erosion, and the erosion dominant factor indicates that the total loss is approximately equal to the particle erosion. Material loss at 45° under each velocity and at 90° under the velocities of 16 m/s and 20 m/s can be treated as erosion–corrosion, while the material loss under other conditions is attributed to erosion. This is because carbon steel is more electrochemically reacted than the stainless-steel surface due to different mass transfer resistances. The passive film on the surface of stainless-steel effectively prevents the transfer of reactants and products, thereby reducing the corrosion of stainless-steel in flowing liquids [4]. Another reason is that the surface is quickly passivated again after the passive film is ruptured by particle impact in the liquid–solid two-phase flow, thereby preventing the progress of subsequent reaction. Such a process lasts only for a fraction of a second [18]. Thus, the effect on the electrochemical reaction of stainless steel surfaces is limited. Therefore, the physical damage caused by particle impacts at such a high flow velocity is the dominant factor in material loss. Moreover, the effect of corrosion on erosion must be considered.

**Figure 10:**
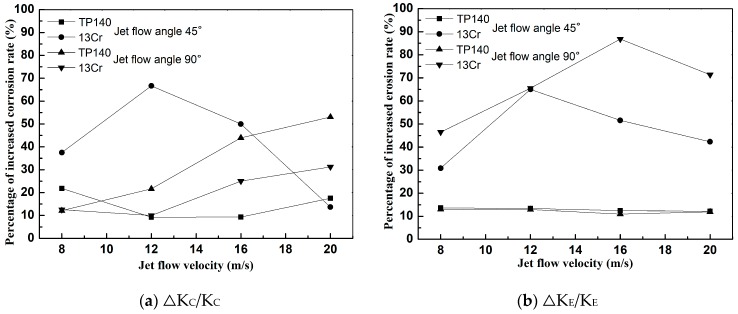
Increased erosion and corrosion rate under liquid–solid and gas–solid or liquid flow: (**a**) increased corrosion rate; (**b**) increased erosion rate.

By comparing the corrosion rates in the liquid and liquid–solid flows (Δ*K_C_*/*K_C_*) and the erosion rates in the liquid–solid and gas–solid flows (Δ*K_E_*/*K_E_*), the percentages of increased erosion and corrosion rate were obtained. The results are shown in Figure 10. Figure 10a shows that the effect of erosion on the corrosion of TP140 and 13Cr increases with flow rate, except in the 13Cr at 45° jet angle, indicating that erosion at high flow velocities effectively enhances corrosion. On the one hand, the increase in flow rate enhances the disturbance by the particles of the boundary layer fluid [19]. On the other hand, high particle impact velocity accelerates the frequency of product film rupture [20]. Figure 10b shows the effect of corrosion on the erosion of TP140 and 13Cr. The erosion growth rate of TP140 is between 10% and 15%, while that of 13Cr increases first and then decreases. Given that particle impact causes fresh metal to be continuously produced on the stainless-steel surface, the effect of corrosion on the erosion in the liquid–solid two-phase flow is significant. Meanwhile, due to the existence of a critical flow rate corresponding to the maximum corrosion rate [21], a critical value for the erosion growth rate of 13Cr steel exists.

**Figure 11:**
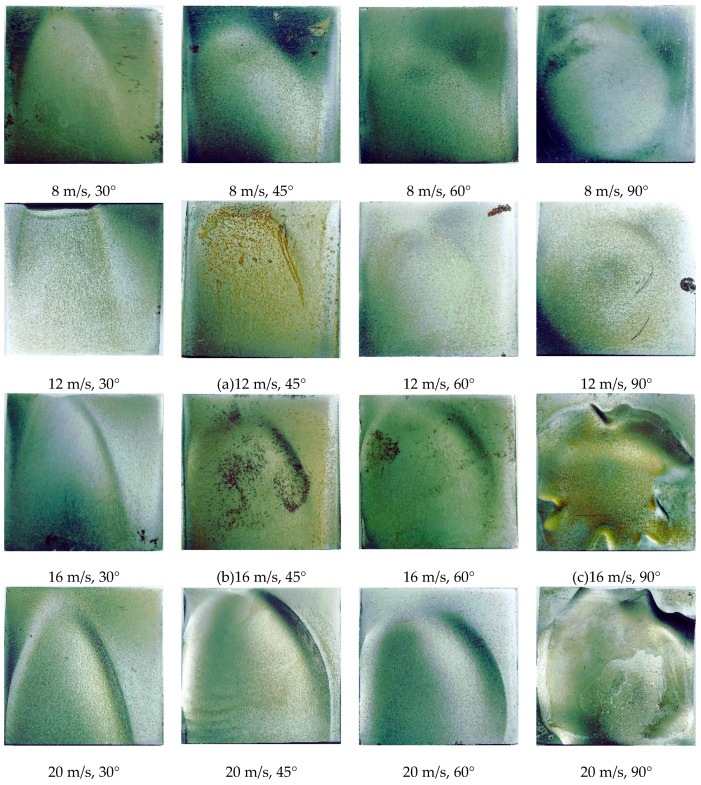
Surface images of 13Cr after erosion and corrosion in liquid–solid two-phase flow.

The erosion surface is susceptible to flow-induced corrosion owing to loose material, large contact areas, and large roughness. Figure 11 shows the erosion images of TP140 at different jet angles and flow velocities. According to Figure 11a–c, the depressed area after erosion is more susceptible to corrosion than the uneroded surface. Corrosion begins to occur near the erosion craters and eventually joins into an area at an angle of 45°, and the erosion areas that are impacted by a large angle can be corroded from craters, platelets, and extruding lips. 

**Figure 12:**
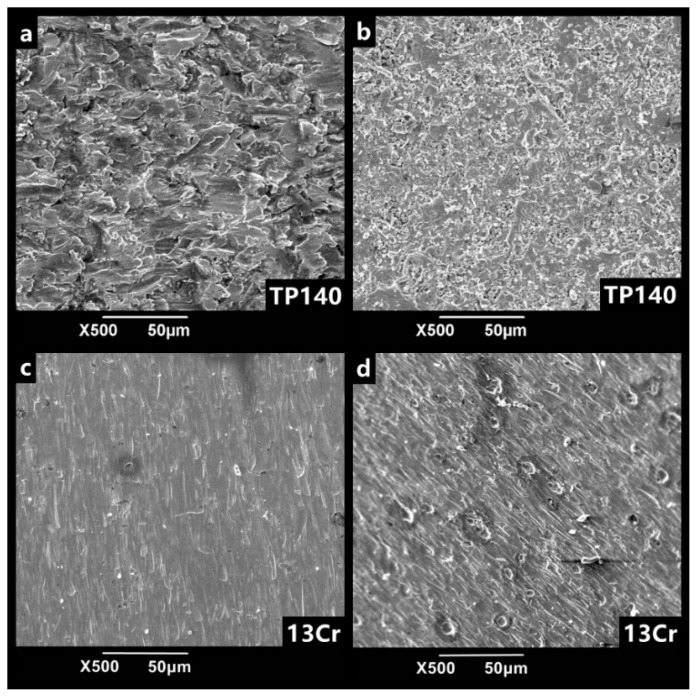
SEM images of erosion surface of TP140 and 13Cr before and after corrosion at the velocity of 8 m/s.

By immersing the erosion surface in flowing liquid for 1 h of corrosion, the surface morphology of 3Cr and TP140 under corrosion and non-corrosion is obtained as shown in Figure 12. The erosion surface of TP140 after corrosion shows that the lip is broken down and changed into platelets (Figure 12b). This is because the corrosion of carbon steel exists in the surface of lips and in the crack, and crevice corrosion reduces the thickness of the root of lips, thereby making the material easier to peel off. At this time, the material is continuously and uniformly peeled off from the surface as the liquid shearing force acts. As for the erosion surface of 13Cr, corrosion does not occur on all surfaces due to the presence of a passivation film instead of the local pits (Figure 12d). Although these pits are not as severe as the uniform corrosion for the matrix, they do not damage the passivation film for the stainless steel. In addition, with the expansion of pitting pits, the erosion resistance of the materials is seriously weakened, especially for the impacts of large particle in high velocity.

## 5. Conclusions

Gas–solid and liquid–solid experiments are used to investigate the erosion–corrosion behavior of the TP140 casing steel and the 13Cr tubing steel. Through experimental measurements and discussion, we draw the following main conclusions:

1. The erosion and corrosion rates of 13Cr and TP140 increase with the flow rate, and a maximum critical angle of erosion exists with the changes in jet angle. The critical angles of TP140 and 13Cr are near 60° and 45°, respectively. In the liquid flow condition, a flow velocity growth increases the current density on the TP140 surface and reduces the corrosion potential of 13Cr, but the effect of the angle on the two parameters is relatively small.
2. Given the rupture of passive film and the reduced passivation rate in NaCl solution, the increased rates of erosion and corrosion of 13Cr are greater than those of TP140. Meanwhile, uniform corrosion and pitting can change the mechanical properties of the metal surface to increase particle erosion. However, the effect of uniform corrosion on erosion is continuously generated, and the effect of pitting will not occur unless it accumulates to a certain extent.
3. Generally, the erosion rate is considerably higher than the corrosion rate in high velocity liquid–solid flow. Therefore, we must pay special attention to corrosion-enhanced erosion, especially for stainless-steel. The experimental results show that the increased erosion rate of TP140 in liquid–solid flow is in the range of 10% to 20%, while that of 13Cr is in the range of 30% to 90% compared to the material erosion rate in the gas–solid phase.

## Figures and Tables

**Table 1:** Chemical composition of TP140 and 13Cr steels (wt %).

Materials	C	Si	Mn	P	S	Cr	Mo	Ni	Cu
TP140	0.26	0.25	0.92	0.007	0.001	1.027	0.423	0.041	0.078
13Cr	0.029	0.22	0.45	0.015	0.001	13.3	1.92	4.85	1.59

**Table 2:** Mechanical properties of TP140 and 13Cr steel.

Materials	Tensile Strength (MPa)	Yield Strength (MPa)	Elongation (%)	Hardness (HV)
TP140	1090	1068	16	287.2
13Cr	970	855	20	323.4

**Table 3:** Erosion and corrosion conditions for jet experiments.

Experiments	Mixed Medium	Jet angle	Jet Flow Velocity
pure erosion	sand + dry air	30°, 45°, 60°, and 90°	8, 12, 16, and 20 m/s
pure corrosion	2.0 wt % NaCl + distilled water	30°, 45°, 60°, and 90°	8, 12, 16, and 20 m/s
erosion-corrosion	2.0 wt % NaCl solution + sand	30°, 45°, 60°, and 90°	8, 12, 16, and 20 m/s

**Table 4:** Summary of experimental measurements of erosion and corrosion components (mm/a).

Jet Flow Angle	Materials	Flow Velocity	*K′_E_*	*K_E_*	Δ*K_E_*	*K′_C_*	*K_C_*	Δ*K_C_*	Δ*K*
45°	TP140	8 m/s	15.93	14.02	1.91	2.4	1.97	0.43	2.34
		12 m/s	18.79	16.57	2.22	2.73	2.5	0.23	2.45
		16 m/s	21.40	19.03	2.37	3.53	3.23	0.3	2.67
		20 m/s	25.73	22.94	2.79	4.29	3.65	0.64	3.43
	13Cr	8 m/s	6.22	4.75	1.47	0.12	0.08	0.04	1.51
		12 m/s	8.42	5.1	3.32	0.15	0.09	0.06	3.38
		16 m/s	13.25	8.74	4.51	0.22	0.14	0.08	4.59
		20 m/s	15.07	10.59	4.48	0.38	0.22	0.16	4.64
90°	TP140	8 m/s	15.13	13.38	1.75	1.11	0.99	0.12	1.87
		12 m/s	17.28	15.3	1.98	1.35	1.11	0.24	2.22
		16 m/s	19.09	17.21	1.88	2.26	1.57	0.69	2.57
		20 m/s	22.82	20.39	2.43	2.51	1.64	0.87	3.30
	13Cr	8 m/s	4.76	3.25	1.51	0.19	0.08	0.11	1.62
		12 m/s	6.90	4.17	2.73	0.23	0.1	0.13	2.86
		16 m/s	9.53	5.1	4.43	0.35	0.12	0.23	4.66
		20 m/s	11.24	6.56	4.68	0.12	0.16	−0.04	4.64

**Table 5:** Four divided regimes of erosion–corrosion.

Dominant Factor	Value
erosion	*K′_C_*/*K′_E_* < 0.1
erosion-corrosion	0.1 ≤ *K′_C_*/*K′_E_* < 1
corrosion-erosion	1 ≤ *K′_C_*/*K′_E_* < 10
corrosion	*K′_C_*/*K′_E_* ≥ 10
